# Predictors of Mortality in Hospitalized African American Covid-19 Cancer Patients

**DOI:** 10.21203/rs.3.rs-1363151/v1

**Published:** 2022-03-22

**Authors:** Suryanarayana Reddy Challa, Gholamreza Oskrochi, Lakshmi G Chirumamilla, Nader Shayegh, Hassan Brim, Hassan Ashktorab

**Affiliations:** Howard University; American University of the Middle East; Howard University; Howard University; Howard University; Howard University

**Keywords:** African Americans, Predictors, Mortality, Hypoalbuminemia, COVID-19, Cancers, Hospitalization, Gastrointestinal disease

## Abstract

**Background:**

Coronavirus disease 2019 (COVID-19) and associated outcomes manifest differently depending on patients’ background and pre-existing conditions. It remains unclear how African Americans with and without cancer have been affected.

**Aim:**

To determine epidemiological, clinical comorbidities, and laboratory test results to identify markers associated with mortality in COVID-19 cancer patients.

**Methods:**

We reviewed all Covid-19 hospitalized patients records from Dec. 2019 to Oct. 2021 at Howard University Hospital. Patients having a history of, or active cancer status were reviewed. All the clinical, treatment, lab values, and pathological data were extracted. Statical analysis of the Covid-19 cancer patients and comparison with non-cancer Covid-19 patients was performed using univariate and multivariate analyses.

**Results:**

Out of 512 COVID-19 infected patients, a total of 49 patients were identified with different types of cancer, with both active and previous history. Females consisted of 26 cancer patients (53%). African American race was predominant in both cases and controls, 83.6% and 66.7% respectively. Cancer patients were older than non-cancer patients (Mean Age-70.6 vs. 56.3 years) and had an increased length of hospital stay (Mean 13.9 vs 9.4 days). Among cancer patients, breast cancer was more prevalent in females and prostate cancer in males, (54% and 52% respectively). Comparison of patients with active vs. previous cancer showed no significant difference in the clinical outcome, death vs. discharge (P=0.34). A higher reduction in albumin level in cancer cases, from the time of admission to day five, was significantly associated with death during the same hospital stay compared to those discharged (n=24, 48.9%, p<0.001). In controls, Lymphopenia (n=436, 94.1%, p=0.05), AST (n=59, 12%, p=0.008) and Albumin (n=40, 10.7%, p=0.02) have shown an association with increased mortality.

**Conclusion:**

Albumin level has shown to have an inverse relationship with clinical outcomes among all COVID infected African American patients. Reduction in Albumin level during the hospital stay, particularly in COVID-19 cancer patients should be considered as a predictor of mortality. No significant difference was noticed in the clinical outcome in patients with previous versus active cancer. Further research with a large cohort size is needed to verify and identify other predictors of outcome in Covid-19 cancer patients and develop appropriate treatment modalities.

## Introduction

In the current pandemic, COVID-19 infection has shown a steady surge with emerging new variants, affecting 300 million and causing 5.5 million deaths globally[1]. It is well known that patients with multiple comorbidities are at high risk of COVID-19 infection with severe disease course and long hospital stays[2]. Cancer patients are immunocompromised from the disease itself or from the treatment which makes them more vulnerable to acquire infections. As per 2018 statistics, around 18 billion cancer cases are present worldwide. The data relating to the impact and clinical outcomes of COVID-19 infection on cancer patients is limited but emerging.

Standard screening of asymptomatic patients plays a vital role in the early diagnosis of various cancers, including colorectal, cervical cancers, breast, and prostate[3]. Nevertheless, the COVID-19 pandemic has caused many institutions to halt screenings, as healthcare providers balance the risk of COVID-19 with waiting weeks or months to screen patients. The unusual burden of COVID-19 on healthcare systems worldwide has significant implications for cancer care. Due to the risk of transmission of the virus, shortage of medical facilities, avoidance of preventive care and overwhelming burden on the healthcare workers, there was a significant delay in patient care. Previous studies have identified cancer as a significant risk factor for severe COVID-19 with increased ICU admission and mechanical ventilation[4]. In a prospective study, COVID-19 patients who had recently undergone cancer treatment had a more increased risk of severe adverse events, possibly due to the immunosuppressive state caused by the use of the cancer treatments such as chemotherapy, radiotherapy, surgery or hormonal therapy[5]. The challenge confronted by physicians treating cancer patients with COVID-19 is not knowing whether to treat or withhold the treatment.

Minorities, particularly African Americans are mostly uninsured and underserved with chronic medical conditions. As per Center for Disease Control (CDC) US Cancer statistics, they also contribute to a large subset of the cancer group with increased incidence of cancers compared to the White, Hispanic or Asian race[6]. Recent studies have shown a 3-to-6-fold increase in the risk of COVID-19 infection among this group, mainly attributed to underlying multimorbidity[7]. So far, there were no studies that have assessed the predictors of morbidity and mortality, among these cancer patients which is critically important to identify and address them timely to decrease mortality. We retrospectively reviewed all the hospitalized COVID-19 cancer patients at our institution, serving a majority of the minority population to determine epidemiological, clinical comorbidities, and laboratory test results to identify markers associated with mortality.

## Methods

### Patients

**Figure 1:**
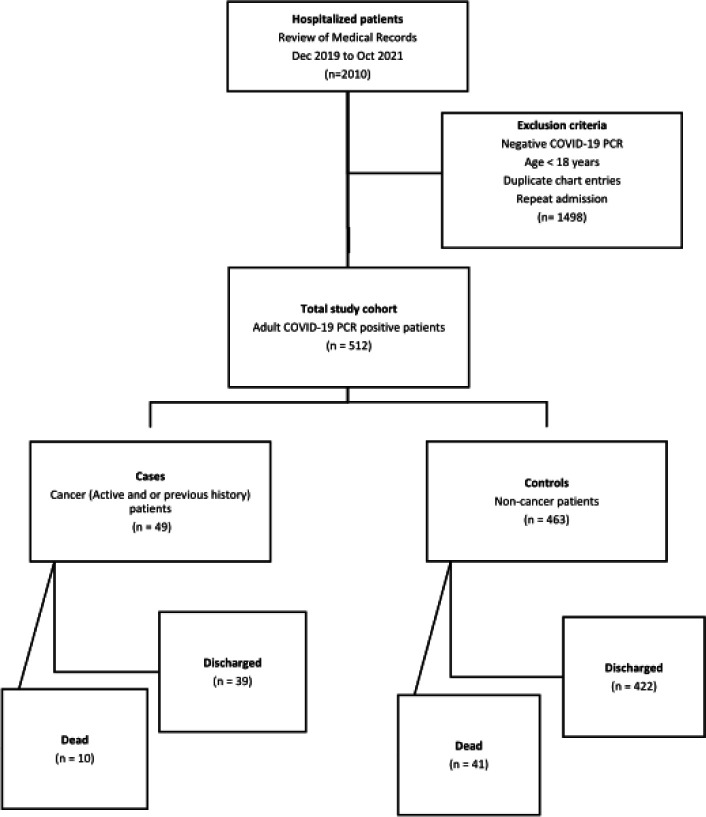
Study flow chart

It is a single-center retrospective case-control study, we analyzed de-identified COVID-19 positive hospitalized patients’ data from Dec. 2019 to Oct. 2021 at Howard University Hospital to determine predictors of mortality in cancer patients (cases) compared to non-cancer patients (controls) (Fig. 1). The chart review of the patients was approved by Howard University Institutional Review Board (IRB) committee (MED-79-12). All the patients were coded in to a excel file and encrypted. All the data and variables streamline and homogenize in the process of data collection and database construction. All the baseline demographics, clinical, lab values and pathological data were extracted from the electronic medical record (EMR) including the type of cancer and time when treatment was received (> 6 months ago, within 6 months or active therapy).

#### Inclusion criteria:

The following criteria were adopted to validate patient selection: patients with a confirmed diagnosis of COVID-19 (PCR positive) who were hospitalized were screened and we identified 512 patients (Fig. 1). Patients with a prior history or current diagnosis of any cancer are considered as cases. All patients were screened for cancer-based on the presence of an International Classification of Diseases, Tenth Revision-ICD-10 diagnostic code in their medical records. Patients without cancer but with a confirmed diagnosis of COVID-19 were included as controls.

#### Exclusion criteria:

The following exclusion criteria were adopted to filter out incomplete data (Fig. 1): Patients where PCR did not confirm COVID-19 diagnosis for controls and cases; Patients below 18 years of age.

### Statistical analysis

Data were analyzed using an Excel spreadsheet and IBM SPSS software. Data were expressed as frequencies and percentages or as mean +/− standard deviation, as appropriate. Covid-19 cancer patients and comparison with controls, non-cancer Covid-19 positive patients were performed using univariate and multivariate analyses. Data from both cases and controls were analyzed separately as well.

## Results

### Patients with active vs. previous cancer showed no significant difference in the clinical outcome

Our study collected data from 512 hospitalized COVID-19 positive patients. Among them, 49 (9.5%) were having a history of or active cancer (Flowchart). Baseline characteristics of cases and control are shown in table 1. Females consisted of 26 (53%) in patients with cancer and 227 (49.1%) in patients without cancer. African American race was predominant in both cases and controls, 83.6% and 66.7% respectively. We have noticed cancer patients were older than non-cancer patients (Mean Age-70.6 vs. 56.3 years) and had an increased length of hospital stay (Mean 13.9 vs 9.4 days). Among cancer patients, breast cancer was more prevalent in females and prostate cancer in males, 54% and 52% respectively. Ten patients with cancer, 2 patients with active and 8 patients with a history of cancer passed away during the same hospital stay. Comparison of patients with active vs. previous cancer showed no significant difference in the clinical outcome, death vs. discharge (P = 0.34; Table 2).

### In both cases and controls, there were no correlation between individual symptoms or clinical comorbidities and death.

Cough (cases: n = 33, 67.3%; control: n = 281, 60.6%) and shortness of breath (cases: n = 30, 61.2% and controls: n = 280, 60.4%) are the most common presenting symptoms in both cases and controls. Hypertension and Diabetes mellitus are the most common pre-existing conditions in both cases and controls. In both cases and controls, univariate and multivariate analyses did not show any effect on the correlation between individual symptoms or clinical comorbidities and death.

### Higher reduction in albumin level in cancer cases associated with death.

A higher reduction in albumin level in cancer cases, from the time of admission to day five (Mean 3.40g/dl to 3.02g/dl), was significantly associated with death during the same hospital stay compared to those discharged (p < 0.001). In controls, a decrease in albumin, low lymphocytes count, and elevated AST showed an independent association with increased mortality, even though statistical significance was not achieved (Table 3).

## Discussion

The severity of COVID-19 infection can range on a spectrum of asymptomatic to critical illness particularly affecting the high-risk groups which include males, older age, and with a history of multiple comorbidities[8]. It is well known by now that Infections are more common in people with cancer, from the disease itself or from the chemotherapy weakening their immune system. Most studies have shown that adults with active cancer, particularly advanced hematologic or lung cancer undergoing active chemotherapy are prone to severe COVID-19 infection[9, 10]. Based on pooled studies, we have observed various laboratory abnormalities in COVID-19 infection but in hospitalized patients progressive decline in lymphocyte count and raise in D-dimer were noted to be associated with worse clinical outcomes[11]. By far, there was less information on assessing the predictors of mortality in COVID infected cancer patients.

Albumin is a protein synthesized by liver contributing 50–60% of total circulating protein in the plasma and is a part of liver function test. Low levels of serum albumin can be secondary to decreased synthesis or increased loss from kidneys, gastrointestinal tract, skin or extracellular space[12]. In the current pandemic, Seow et al analyzed single-cell RNA sequencing in human liver tissues and identified co-expression of Angiotensin-Converting Enzyme 2 (ACE-2) and Transmembrane Serine Protease 2 Expression (TMPRS) in the Liver progenitor cells, which serves as entry receptor for COVID-19 Spike (S) protein resulting in Liver injury and associated abnormalities like hypoalbuminemia and elevated liver enzymes[13].

In hospitalized patients, Akirov et al showed that there was with an increased risk of mortality with marked hypoalbuminemia (< 2.5g/dl), 34% compared to mild hypoalbuminemia (2.5–3.5g/dl) or normal albumin level (> 3.5g/dl) on admission, 12% vs 2% mortality respectively. Also noted that normalization of albumin level before discharge was associated with better short- and long-term survival[14]. Similarly in a systemic review by Gupta et al, assessing different types of gastrointestinal tract cancer patients, pretreatment low serum albumin levels were significantly association with mortality and have not been any different from general hospitalized patients[15].

In a retrospective analysis by Viana Llamas et al, on COVID-19 infected patients, hypoalbuminemia on admission was observed in 66% of non-survivor’s vs 38% in survivors[16] and in another multicenter study by Turcato et al, albumin level < 3.5g/dl in COVID-19 patients was shown to be associated with severe COVID infection developing sepsis and 30-day mortality [17]. In our study cohort, we have also noticed hypoalbuminemia is associated with mortality in both cancer and non-cancer subjects. But one important observation we have come across is, progressive decline in albumin level, from admission to day 5, in COVID-19 infected cancer patients was significantly associated with death during the same hospital stay.

Apart from hypoalbuminemia, COVID-19 infected patients presenting with gastrointestinal symptoms have abnormal liver associated enzymes, as noticed by Wang et al in 2.6–53% of patients, with higher levels observed in non-survivors[18]. In our study cohort, controls patients have shown low albumin level, and elevated liver enzymes, even though not statistically significant are each individually associated to mortality.

A multivariate analysis by Williamson et al, assessed primary care records of 17 million people, with majority of White population, 63% in England have shown that Black and South Asian group, contributing 8% of the study group has slight increased risk of COVID-19 related death compared to White ethnicity, hazard ratio (HR) 1.48 and 1.45 respectively. From the same study, they have also noticed that hematological malignancies carry a higher risk of death in COVID-19 infected patients compared to nonhematological malignancies, 4-fold vs 1.8-fold respectively[19]. In our study, there was no significant mortality difference noticed based on the status, active vs previous history of cancer or type of cancer which could probably be attributed to large number of hospitalized African Americans, who are underserved with lack of proper primary care and follow ups, suffering from multiple comorbidities at baseline plus there was also a difference in the prevalence of cancer type.

So far, the management of hospitalized cancer patients with mild or severe COVID-19 infection is the same as that used for the general population. There is limited knowledge on factors that need to be addressed to decrease mortality in COVID-19 infected cancer patients. Based on our observation, reviewing all the available demographic, clinical and laboratory variables in our cohort, we strongly suggest that low albumin level on admission or progressive decline during the hospital stay should not be neglected and advise further research to see if normalizing the levels will have any impact on survival of the COVID infected cancer patients.

The smaller number of cancer patients in our study group has limited the capability to identify an association between symptoms on admission, vitals, and underlying comorbidities with mortality. Due to the same, the most common complication or a casualty of death was not reliably reported. We analyzed only hospitalized patients, so we do not have information on outpatients. Our study group included patients from pre and post vaccination time period so, we don’t have the information on the COVID-19 vaccination status for each individual, which if present could have provided some insights on its effect on cancer patients that were discharged vs diseased.

In conclusion, albumin level has shown to have an inverse relationship with clinical outcomes among all COVID infected African American patients. Reduction in Albumin level during the hospital stay, particularly in COVID-19 cancer patients should be considered as a predictor of mortality. No significant difference was noticed in the clinical outcome in patients with previous versus active cancer. Further research with a large cohort size is needed to verify and identify other predictors of outcome in Covid-19 cancer patients and develop appropriate treatment modalities

## Figures and Tables

**Table 1:** Baseline characteristics for cases and controls

		Covid-19 Patients with active or history of Cancer (n = 49) n (%)	Covid-19 patients without active/ history of Cancer (n = 463) n (%)
**Age (Mean, years)**		70.6	56.3
**Sex**	Male	23 (47)	236 (50.9)
	Female	26 (53)	227 (49.1)
**Race**	African American	41 (83.6)	309 (66.7)
	White	6 (12.2)	27 (5)
	Hispanic/Latino	0 (0)	111 (23.9)
	Asian	0 (0)	2 (0.4)
	Others/unknown	2 (0.4)	14 (3)
**Length of Hospital stay**		13.9	9.4
**BMI**	Normal	9 (18.3)	98 (21.1)
	Underweight	1 (0.2)	15 (3)
	Overweight	17 (34.6)	138 (29.8)
	Obese	20 (40.8)	169 (36.5)
	Unknown	2 (0.4)	43 (9)
**Comorbidities**	Hypertension	39 (79.5)	240 (51.8)
	Diabetes Mellitus	16 (32.6)	168 (36.2)
	CAD	8 (16.3)	87 (18.7)
**Symptoms**	Fever	14 (28.5)	251 (54.2)
	Cough	33 (67.3)	281 (60.6)
	Shortness of breath	30 (61.2)	280 (60.4)
	Ageusia	5 (1.0)	30 (6)
	Loss of appetite	13 (26.5)	112 (24.1)
	Myalgia	8 (16.3)	97 (20.9)
	Fatigue	10 (20.4)	138 (29.8)
	Abdominal Pain	4 (0.8)	65 (14.0)
	Diarrhea	5 (1.0)	86 (18.5)
	Vomiting	3 (0.6)	53 (8.4)
**Labs elevated**	Ferritin	17 (34)	236 (50.9)
	D-Dimer	42 (85.7)	335 (72.3)
	Creatinine	36 (73.4)	179 (38.6)
	Bilirubin	6 (12)	11 (2)
	AST	13 (26)	59 (12)
	ALT	7 (14)	34 (7)
**Labs decreased**	Albumin	28 (57.1)	40 (10.7)
	Lymphocyte	29 (59.1)	436 (94.1)
**ICU admission**		15 (30.6)	104 (22.4)
**Mechanical Ventilation**		10 (20.4)	54 (11.6)
**Death during same hospital stay**		10 (20.4)	41 (8.9)

**Table 2:** Clinical characteristics of cancer patients exposed to COVID-19

		Alive (n = 39)	Dead (n = 10)	P-value
**Type of cancer**	Brain cancer	1	0	
	Breast cancer	12	2	
	Lung cancer	4	1	
	Liver cancer	2	0	
	Kidney cancer	3	1	
	Colon cancer	2	1	
	Multiple myeloma	1	1	
	Leukemia	1	0	
	Prostate cancer	11	1	
	Head and Neck cancer	1	0	
	Brain & Thyroid cancer	1	0	
	Breast cancer & Hodgkin’s lymphoma	0	1	
	Kidney & Lung cancer	0	2	
**Cancer status**	Active	13	2	= 0.344
	Previous history of cancer	26	8	= 0.344

**Table 3:** Predictors of Mortality in hospitalized Cancer patients exposed to Covid-19

		n (%)	P-value
**Cases**	Albumin - day of admission to day 5	24 (48.9)	< 0.001
**Controls**	Elevated AST	59 (12)	= 0.008
	Low lymphocyte	436 (94.1)	= 0.05
	Low albumin	40 (10.7)	= 0.019
